# Phases of Evolution and Heredity

**Published:** 1911-09

**Authors:** 


**Phases of Evolution and Heredity.** By DAVID BERRY HART, M.D., F.R.C.P.E. Pp. xi, 259. London: Rebman Ltd. 1910.

Dr. Berry Hart approaches the subject of heredity with the object of examining the weak points in the mechanism of evolution as described by Darwin.

He points out, in Weismann's terms, that the power of variation lies probably in the gamete and not in the soma, and he then quotes Mendelism as explaining the ratio in which characteristics are inherited.

The author suggests an ingenious modification of the Mendelian scheme which illustrates logically the origin of the Mendelian ratio 1 : 2 : 1.

Next he discusses biometry as founded in Britain by Galton and as advanced by Karl Pearson, and he attributes great credit to the work of these men.

To Semon, the author of *Mnemism,* Dr. Hart refers without immoderate criticism, but he suggests that the engram theory is as hypothetical as any of the suppositions of scholastic philosophy.

He next proceeds to describe what is meant by mutation, but he makes no original suggestions as to its causation.

Having thus shortly surveyed the more salient points in the teachings of the different schools of heredity, Dr. Hart goes on in Chapter VII to the important question of heredity in disease, and in this connection he finds consolation in the "purity of the strain in the germ cell and its non-affection by the soma," which accounts for the fact that many diseases formerly thought to be hereditary are not so.

This little book constitutes a short survey of the views which have been expressed by some of the leading authorities on this subject of heredity.

